# Effects of transcutaneous auricular vagus nerve stimulation or combined vagal and trigeminal nerve stimulation on platelet function and laboratory hemostasis parameters in healthy human subjects

**DOI:** 10.1186/s42234-026-00208-w

**Published:** 2026-06-05

**Authors:** Jared M. Huston, Carlos E. Bravo-Iñiguez, Julien Papoin, Maaryj Ahmad, Brooke Le, Isabella Mirro, Melanie McWade, Caroline Benner, Alejandro Covalin, Christopher J. Czura, Navid Khodaparast, Lionel Blanc

**Affiliations:** 1 Department of Surgery, Northwell Health, 2000 Marcus Avenue, New Hyde Park, NY 11042 USA; 2 Department of Science Education, Donald and Barbara Zucker School of Medicine at Hofstra/Northwell, Hempstead, NY 11549 USA; 3 Institute of Bioelectronic Medicine, Feinstein Institutes for Medical Research at Northwell Health, 350 Community Drive, Manhasset, NY 11030 USA; 4 Elmezzi Graduate School of Molecular Medicine at Northwell Health, 350 Community Drive, Manhasset, NY 11030 USA; 5 Institute of Molecular Medicine, Feinstein Institutes for Medical Research at Northwell Health, Manhasset, NY 11030 USA; 6 Department of Pediatrics, Donald and Barbara Zucker School of Medicine at Hofstra/Northwell, Hempstead, NY 11549 USA; 7 Spark Biomedical Inc., Dallas, TX 75252 USA

**Keywords:** Vagus nerve stimulation, Trigeminal nerve stimulation, Neuromodulation, Bleeding, Hemorrhage, Hemostasis, Trauma, Surgery, Platelets, Spleen

## Abstract

**Background:**

Traumatic or surgical hemorrhage causes substantial morbidity and mortality. Electrical vagus nerve stimulation (VNS) reduces traumatic hemorrhage in animal models. VNS targets acetylcholine-producing T lymphocytes in the spleen to increase intracellular calcium within circulating platelets via α7 nicotinic acetylcholine receptors. Elevated calcium levels facilitate platelet activation (priming) after tissue injury to accelerate and increase clot formation that improves hemostasis. Trigeminal nerve stimulation (TNS) also decreases traumatic hemorrhage in mice, but the mechanism remains unknown. Recently, we showed that transcutaneous auricular neurostimulation (tAN; combined auricular VNS and TNS) reduces blood loss and days of menstruation in women with idiopathic or von Willebrand disease related heavy menstrual bleeding. The ability of tAN or transcutaneous auricular VNS (taVNS) to improve platelet function or laboratory hemostasis remains unknown.

**Methods:**

Here we performed a prospective, randomized, double-blind, sham-controlled, single-center, first-in-human exploratory trial to determine the safety and efficacy of taVNS or tAN to prime platelets and augment clot formation. Healthy adult subjects received sham stimulation before taVNS or tAN, followed by serial measurements of platelet and hemostasis markers, including platelet functional analysis, thrombin generation, blood counts, coagulation assays, and thromboelastography. Repeated measures one-way ANOVA followed by Bonferroni’s test was used for comparisons between three or more time points. Two-tailed paired T-test was used for comparisons between two time points.

**Results:**

Administration of taVNS or tAN was well tolerated without observable adverse events during the study period. taVNS or tAN primed platelets via collagen- or ADP-mediated signaling pathways, respectively. taVNS accelerated clot initiation, propagation, and stabilization as measured by thromboelastography. There were no differences in systemic or local thrombin generation, circulating white or red blood cell counts, platelet counts, prothrombin time, partial thromboplastin time, or INR assays after administration of taVNS or tAN.

**Conclusions:**

These results provide evidence that taVNS or tAN primes human platelets and taVNS accelerates clotting kinetics as quantified by thromboelastography. taVNS and tAN warrant additional clinical study as therapies for traumatic or surgical hemorrhage and congenital or acquired coagulopathies.

**Trial registration:**

This study is registered with the ClinicalTrials.gov database (http://clinicaltrials.gov). The registration number is NCT05977946. The study start is 10–31-2023.

**Supplementary Information:**

The online version contains supplementary material available at 10.1186/s42234-026-00208-w.

## Background

Hemostasis, or “halting of blood”, is critical for survival (Davidson et al. 2003). Depictions of the Battle of Troy in Greek mythology indicate humans recognized the lethality of exsanguination long ago (Santos 2000). Not surprisingly, wound styptics arose in Ancient Greece, (Harvey 1929) and the military campaigns of Alexander the Great describe fitting mechanical tourniquets on extremity wounds (MS 2014). Current trauma care utilizes similar techniques, including kaolin-impregnated gauze and advanced tactical field tourniquets (Peng 2020). Despite these advances, life-threatening hemorrhage persists. Bleeding is the most common preventable cause of death after civilian trauma, (Drake et al. 2020) and non-compressible torso hemorrhage is the leading cause of battlefield mortality (Eastridge et al. 2012; Martin et al. 2009). Modern surgery, akin to controlled tissue trauma, carries significant risk of bleeding despite skilled surgeons armed with state-of-the-art technologies (Roshanov et al. 2021). Even caesarian section, the most common surgical procedure worldwide, is plagued by life-threatening bleeding (Practice Bulletin No 2017). As such, post-partum hemorrhage is a leading cause of maternal deaths (Say et al. 2014).

Current strategies to reduce hemorrhage share fundamental limitations. Because they are deployed after bleeding onset, precious time (and blood) are wasted, resulting in higher mortality (Harmsen et al. 2015). Topical gauze and extremity tourniquets are ineffective against thoracic or abdominal bleeding. Mechanical or non-specific pharmacological agents that target non-compressible hemorrhage risk blood vessel injury, tissue ischemia, and distant clot formation (Chen et al. 2024; Kam et al. 2001; Tran et al. 2024). Perhaps most problematic, however, is that therapies fail to harness platelets, the body’s primary systemic hemostatic effector cells. In contrast, nearly a dozen medications, some in use for decades, inhibit platelets and clotting in coronary artery disease, cerebrovascular disease, and other hypercoagulable states (Iqbal 2025). There are no clinically approved pharmacological approaches to stimulate platelets to improve clotting and reduce bleeding.

Bioelectronic medicine cures disease by modulating electrical activity within the nervous system (Huston and Tracey 2011; Pavlov and Tracey 2015). Since 1997, implanted cervical vagus nerve stimulation (VNS) has treated medically refractory epilepsy and depression (Wheless et al. 2018). Recently, VNS was FDA approved for chronic ischemic stroke and rheumatoid arthritis, and it is undergoing clinical trials for Crohn’s disease (D'Haens et al. 2023; Peterson et al. 2024; Deng et al. 2025). Previously, we showed that VNS accelerates clot initiation, increases clot deposition at injury sites, and reduces blood loss after peripheral soft tissue trauma in swine (Czura et al. 2010). We elucidated this hemostatic mechanism using murine hemorrhage and thrombosis models (Bravo-Iñiguez et al. 2023). VNS harnesses acetylcholine-producing T-lymphocytes contacting circulating platelets in the spleen (Bravo-Iñiguez et al. 2023). Cholinergic signaling via platelet α7 nicotinic acetylcholine receptors increases intracellular calcium that facilitates (primes) secretion of prothrombotic α granules (Bravo-Iñiguez et al. 2023). VNS-primed systemic platelets augment clot formation to decrease bleeding (Bravo-Iñiguez et al. 2023). Subsequent work in our laboratory showed that electrical trigeminal nerve stimulation (TNS) reduces murine traumatic hemorrhage (unpublished). Most recently, we reported that transcutaneous auricular neurostimulation (tAN; combined VNS and TNS) reduces blood loss and days of menstruation in women with idiopathic or von Willebrand disease (vWD) related heavy menstrual bleeding (Czura et al. 2025). Considering these findings, we reasoned that transcutaneous auricular VNS (taVNS) or tAN can prime systemic platelets and improve laboratory hemostasis in healthy subjects. We administered sham stimulation followed by taVNS or tAN to 24 healthy subjects to determine the effects on systemic platelet activation, thromboelastography, coagulation assays, blood counts, and systemic and local thrombin generation.

## Methods

This study is registered with the ClinicalTrials.gov database (http://clinicaltrials.gov). The registration number is NCT05977946.

### Trial design

**Fig. 1:**
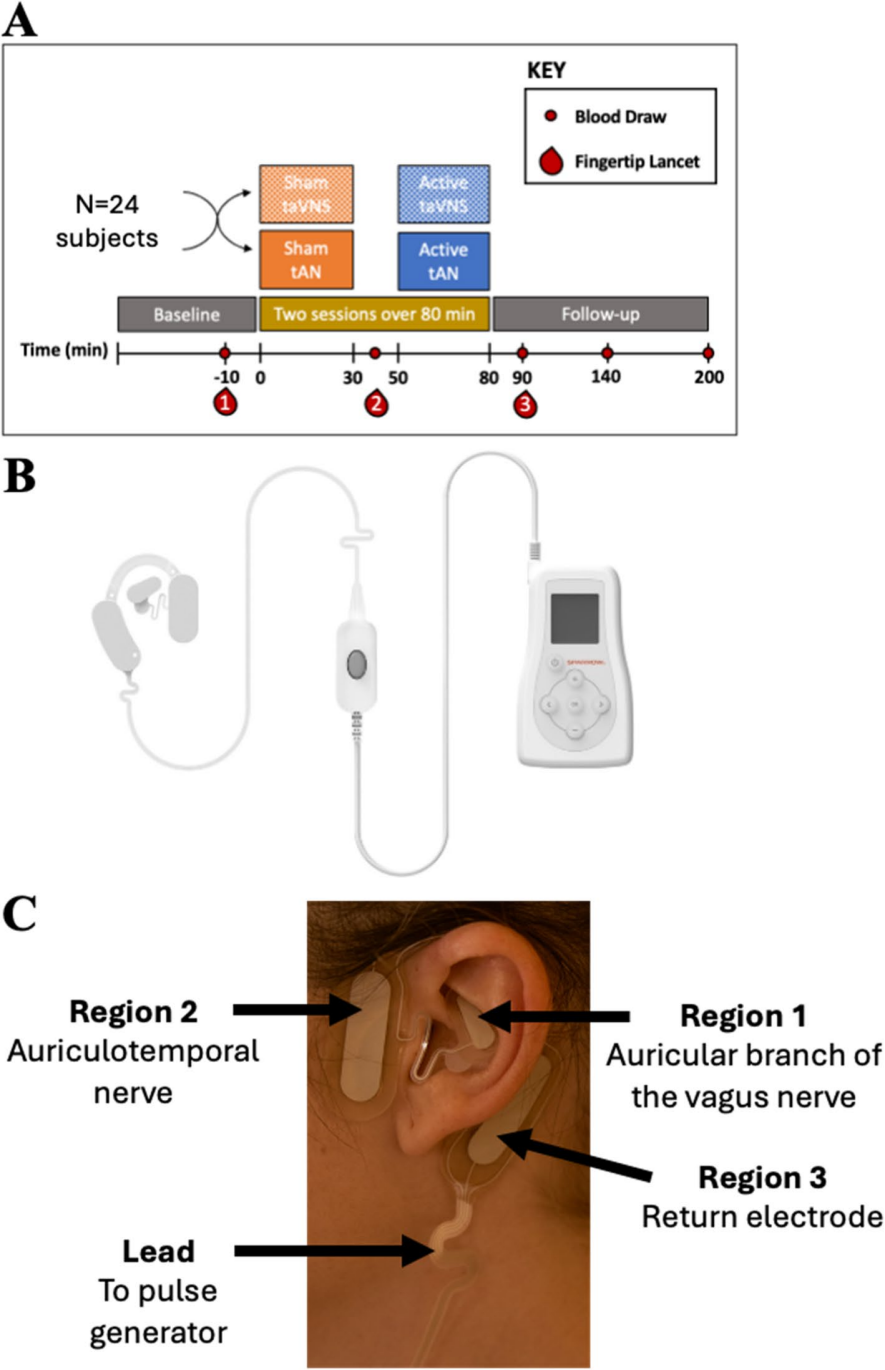
Trial design and interventions. **A** After randomization, subjects underwent baseline blood draws followed by sham taVNS or tAN for 30 min, post-sham blood draws after 10 min, active taVNS or tAN for 30 min, and follow-up blood draws after 10 min, 1 h, and 2 h. **B** Transcutaneous auricular VNS (taVNS) of auricular branch of the vagus nerve (ABVN) or tAN (stimulation of auriculotemporal nerve (ATN) and ABVN) administered via Spark Volta device. **C** Electrode positioning for taVNS (regions 1 and 3) or tAN (regions 1, 2 and 3). Region 3 is the return electrode. Adapted from Czura et al. (Czura et al. 2026)

This study was a prospective, randomized, double-blind, sham-controlled, single-center exploratory trial with two study arms; Group 1 included sham taVNS of the auricular branch of the vagus nerve (ABVN) followed by active taVNS; Group 2 included sham tAN of the ABVN and auriculotemporal nerve (ATN), a sensory branch of the trigeminal nerve, followed by active tAN (Fig. 1A). This study was approved by the WCG™ institutional review board. All enrolled participants provided written informed consent. All research procedures were performed in accordance with guidelines set forth by the Feinstein Institutes for Medical Research at Northwell Health (Manhasset, NY USA).

### Participants

Adult subjects were screened for enrollment after obtaining consent. Inclusion criteria included the following: 1) ages 18 to 65; 2) English proficient; 3) able to provide informed consent and comprehend all study requirements. Exclusion criteria included the following: 1) history of thrombocytopenia, coagulopathy, abnormal bleeding or easy bruising; 2) history of anemia or anemia-related disorders; 3) history of stroke, pulmonary embolism, myocardial infarction, or deep venous thromboses; 4) use of coagulation- or platelet-modifying therapies; 5) history of chronic tobacco use or nicotine ingestion in the past three months; 6) caffeine consumption within 12 h; 7) blood transfusion within 30 days; 8) history of seizures, neurologic diseases, or traumatic brain injury; 9) presence of devices (e.g., pacemakers, neurostimulators); 10) abnormal ear anatomy or infection; 11) females of childbearing potential, not using adequate contraception, or unwilling to use contraception for the study; 12) pregnant, lactating or menstruating females; 13) significant disease that may put the participant at risk due to participation or influence trial results or subjects ability to participate. Any concerns on the part of the research coordinator related to potential subjects meeting inclusion or exclusion criteria prompted further review by the principal investigator before proceeding with study enrollment.

### Randomization

Subjects were allocated in a 1:1 ratio to either treatment arm (tAN or taVNS) by the study sponsor, based on a National Cancer Institute clinical trial randomization tool (https://ctrandomization.cancer.gov/tool). The randomization method was asymptotic maximal into two arms with no stratification, and a maximum tolerated imbalance (MTI) of 3 (Zhao et al. 2018). The MTI occurred once after the penultimate subject. As such, the final subject was non-randomly assigned to the opposite study group. Participant cohort assignment was provided to the site clinical study research coordinator at study onset.

### Blinding

The subjects and study team were blinded to the treatment arms. The research coordinator was unblinded to perform randomization and apply the assigned stimulation. The research coordinator did not discuss group allocations with the study team. Earpiece stimulator design was identical for tAN or taVNS; the anatomical site of stimulation was determined by the Patient Controller. All participants were blinded to group assignment and timing of active versus sham stimulation. Participants were informed that they may perceive stimulation, and that they may receive benefits without any perception of stimulation.

### Interventions

Following enrollment and randomization, participants underwent baseline blood draw via an IV line and fingertip lancet blood collection (Fig. 1A). The left ear was cleaned and fitted with the Volta earpiece (Fig. 1B) connected to a Patient Controller programmed using the Clinical Tool application to deliver sham taVNS or sham tAN for 30 min. After ten minutes, participants underwent systemic blood draw and fingertip blood collection. After ten minutes, participants received 30 min of active taVNS or tAN. taVNS parameters included a pulse width of 250us and frequency of 30 Hz in region 1 (ABVN) (Fig. 1C). tAN parameters included a pulse width of 250us and frequency of 30 Hz in region 1 (ABVN) and 100 Hz in region 2 (ATN) (Fig. 1C). Region 3 served as return electrode for taVNS and tAN (Fig. 1C). The duty cycle was 5 min with a 10-s off period. Stimulation intensities were set initially to 2.5 mA for both regions. If participants reported discomfort, the intensity was decreased in increments of 0.1 mA until reaching a comfortable level. Participants underwent systemic blood draws 10 min, 1 h, and 2 h after stimulation completion. Fingertip blood collection occurred at the 10-min time-point. At study conclusion, a questionnaire was administered to determine if participants perceived a sensation of stimulation during the sessions.

### Outcomes

The performance measures included changes in systemic markers of hemostasis (thromboelastography with platelet mapping, prothrombin time, partial thromboplastin time, international normalized ratio, TAT complex concentrations, D-dimer); changes in platelet activation markers as determined by flow cytometry; changes in local shed blood TAT complex concentrations; and circulating blood cell counts. Safety was assessed by routine adverse event reporting throughout the study.

### Sample size

As an exploratory study, no formal power analysis was performed. Based on prior literature, the estimated sample size suggested three subjects per group were required to show differences in bleeding time following stimulation. However, the estimated sample size for determining differences in blood biomarkers was 10–12 subjects per group. These results are based on 24 subjects who completed all phases of the study.

### Statistical methods

All data were presented as mean ± SEM unless noted otherwise. Fisher’s exact test was used for comparisons between subject sex, race, and ethnicity. Two-tailed Mann Whitney test was used for comparison of subject ages. Repeated measures one-way ANOVA followed by Bonferroni’s multiple comparisons test was used to compare data collected over three or more time points. Two-tailed paired T-test was used to compare data collected over two time points. A *p* ≤ 0.05 was considered statistically significant. Statistical analyses were performed, and data were presented using GraphPad Prism 10 (GraphPad Software), BD FACSDiva software (BD Biosciences), and FlowJo 10 (LLC). Random results from nine TEG assays (four tAN and five taVNS subjects) were performed outside of the manufacturer’s recommended analysis window (> 4 h after blood collection) and were excluded from final analysis. Two subjects received the incorrect stimulation paradigm but were included in the intent-to-treat analysis. No adjustments to datasets were made for missing datapoints.

### Cell counts

Whole blood was collected from a peripheral vein and placed into standard EDTA-coated tubes. Complete blood counts (CBC) were measured using an automated hematology analyzer (Advia 120 multispecies hematology analyzer, Bayer Healthcare).

### Thromboelastography, platelet mapping, and coagulation assays

Samples of whole blood were collected from a peripheral vein and placed into glass blood collection tubes with sodium citrate and the TEG® 6 s Hemostasis system (Haemonetics Corporation, Boston, MA, USA) was used to generate coagulation profiles. The TEG® 6 s utilized PlateletMapping® cartridges to produce a qualitative assessment of platelet function. Prothrombin time (PT), partial thromboplastin time (PTT), international normalized ratio (INR), and D-dimer were measured according to routine clinical pathology laboratory procedures.

### Platelet receptor analysis

Platelet cell surface markers were assessed as described previously (Bravo-Iñiguez et al. 2023). Systemic blood was collected and placed into glass blood collection tubes with ACD solution. Whole blood was diluted with modified Tyrode’s Buffer (134 mM NaCl, 0.34 mM Na2HPO4, 2.9 mM KCl, 12 mM NaHCO3, 20 mM Hepes, pH 7.0 with 5 mM glucose, 0.35% BSA) and stimulated ex vivo with thrombin (1U/ml, Sigma-Aldrich), collagen (5 μg/ml, Sigma-Aldrich), or ADP (20 μg/ml, Thermo Scientific). Samples were stained with mouse anti-human monoclonal antibodies against CD42b (BUV563, BD OptiBuild 741,372), CD62P (P-Selectin BV421, BDHorizon 564,038), PAC-1 (FITC, BD 340507), and CD63 (B-V711, OptiBuild 740,800) (Supplemental Fig. 1). Flow cytometry data were collected using the BD FACSymphony™ A3 Cell Analyzer and FACSDiva 8.0 software and analyzed with FlowJo 10.10.0 (Ashland, OR).

### Thrombin generation

Peripheral vein systemic blood or shed blood from finger lancet injury was collected directly into heparinized tubes. Blood was centrifuged for 15 min at 1500 g and plasma stored at − 80 °C until assay. Thrombin generation was determined by measurement of thrombin-antithrombin (TAT) complex via commercially available ELISA (AB108907 Abcam, Cambridge, MA).

## Results

### Subject enrollment and allocation

25 subjects were assessed for eligibility between November 2023 and August 2024. 24 met inclusion criteria and were randomized to tAN (*n* = 11) or taVNS (*n* = 13). No subjects terminated study involvement before completion. 24 subjects were analyzed for laboratory marker endpoints based on intention to treat. A flow diagram detailing enrollment and allocation can be found in (Supplemental Fig. 2). Recruitment was halted after the first 24 subjects due to a performance site operational issue that precluded timely study completion. Laboratory operational issues precluded analysis of some planned blood chemistries and TEG assays; after the first 9 participants completed the study, blood sample collections were reduced from 5 to 2 timepoints (10 min post-sham and 10 min post taVNS or tAN).

### Baseline demographics

The average age of tAN subjects (30.1 ± 7.5 years) was significantly less than taVNS subjects (39.9 ± 12.7 years). There were no significant differences in sex, race, or ethnicity between treatment groups (Table 1).

**Table 1:** Baseline demographics of subjects

**Treatment Group**		**tAN**	**taVNS**
Subjects, *n*		11	13
Mean age and (range), *years*		30.1 ± 7.5 (18–48)*	39.9 ± 12.7 (29–62)*
Female Sex, *n (%)*		10 (91)	9 (69)
Race, *n (%)*			
	White	6 (55)	4 (31)
	Black	1 (9)	2 (15)
	Asian	3 (27)	4 (31)
	Mixed	1 (9)	0 (0)
	Other	0 (0)	3 (23)
Ethnicity, *n (%)*			
	Hispanic	1 (9)	3 (23)
	Non-Hispanic	10 (91)	10 (77)

Data are mean ± standard deviation or n (%). All *p* values are outcomes of Fisher’s exact test or two-tailed Mann Whitney tests

Multiracial (mixed) participant identified as White and Black

^*^ *p* ≤ 0.05

### Effects of tAN or taVNS on circulating blood counts and clinical coagulation parameters

To determine the effects of tAN or taVNS on laboratory hemostasis, we first measured circulating blood cell counts. Compared with sham stimulation, there were no differences in white blood cell count (WBC) after tAN (Supplemental Fig. 3A) or taVNS (Supplemental Fig. 3B). There were no differences in hemoglobin (Hb) after tAN (Supplemental Fig. 3C) or taVNS (Supplemental Fig. 3D). There were no differences in hematocrit (HCT) after tAN (Supplemental Fig. 3E) or taVNS (Supplemental Fig. 3F). Finally, there were no differences in platelet counts following tAN (Supplemental Fig. 3G) or taVNS (Supplementary Fig. 3H). Next, we measured prothrombin time (PT) and international normalized ratio (INR) to assess extrinsic clotting cascade activity, partial thromboplastin time (PTT) to measure intrinsic clotting cascade activity, and D-dimer levels reflecting elevations in systemic clot formation and lysis.

**Fig. 2:**
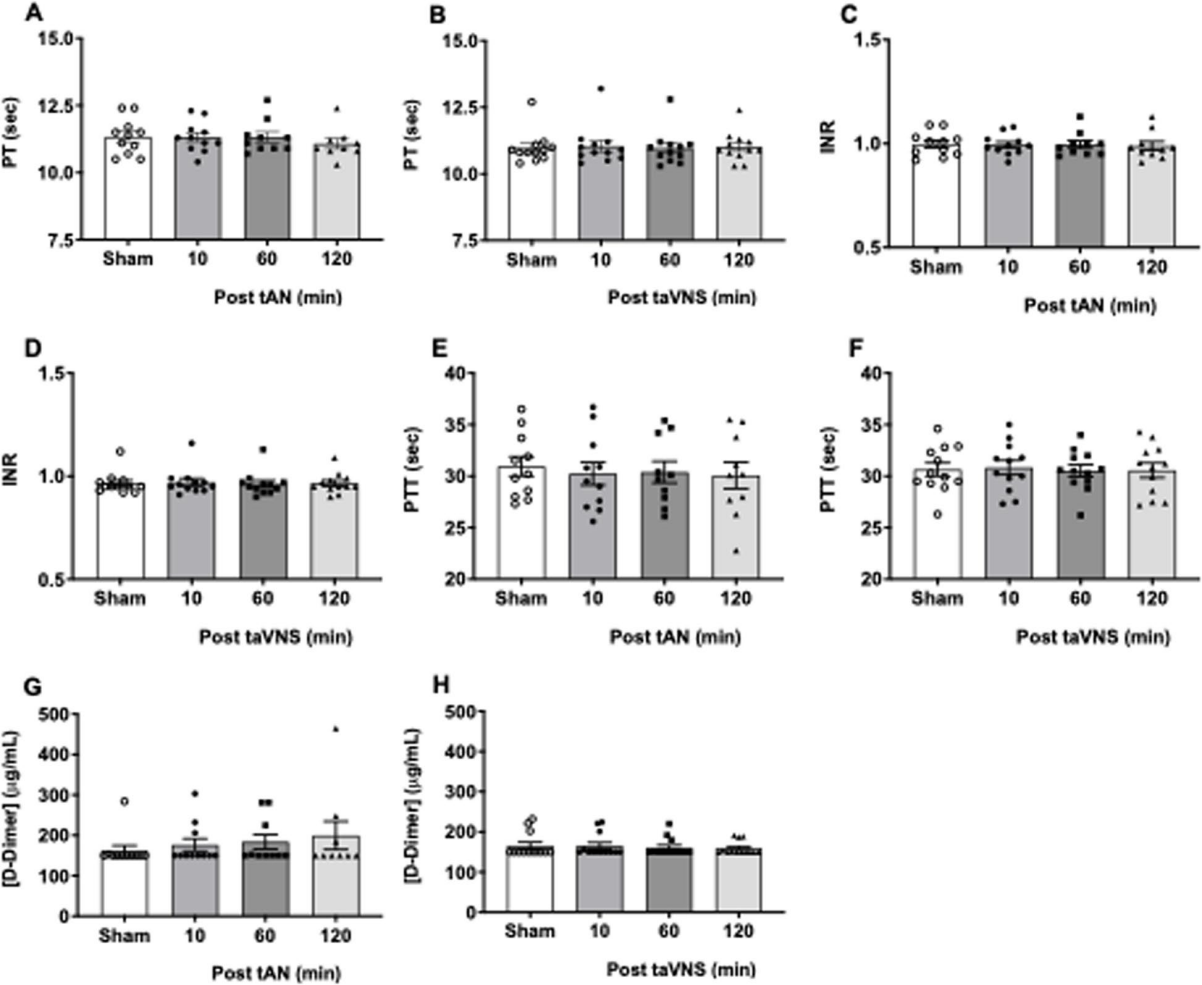
Systemic coagulation profiles following tAN or taVNS. Peripheral circulating blood collected 10 min after sham-stimulation or 10, 60, and 120 min after tAN or taVNS. **A**-**B** Prothrombin time (PT) after tAN (*n* = 11) or taVNS (*n* = 12). **C**-**D** International normalized ratio (INR) after tAN (*n* = 11) or taVNS (*n* = 12). **E**–**F** Partial thromboplastin time (PTT) after tAN (*n* = 11) or taVNS (*n* = 12). **G**-**H** D-dimer after tAN (*n* = 11) or taVNS (*n* = 13). Error bars represent standard error of the mean. P values are compared to post-sham stimulation values. * *p* ≤ 0.05 by repeated measures ANOVA followed by Bonferroni’s multiple comparisons test

Compared with sham stimulation, there were no differences in PT after tAN (Fig. 2A) or taVNS (Fig. 2B). We found no differences in INR after tAN (Fig. 2C) or taVNS (Fig. 2D). There were no differences in PTT after tAN (Fig. 2E) or taVNS (Fig. 2F). Finally, there were no differences in D-dimer levels after tAN (Fig. 2G) or taVNS (Fig. 2H). These data suggest that neither tAN nor taVNS modulates circulating blood counts or extrinsic or intrinsic clotting cascade function or increases systemic hypercoagulability.

### Effects of tAN or taVNS on systemic and local clot formation

**Fig. 3:**
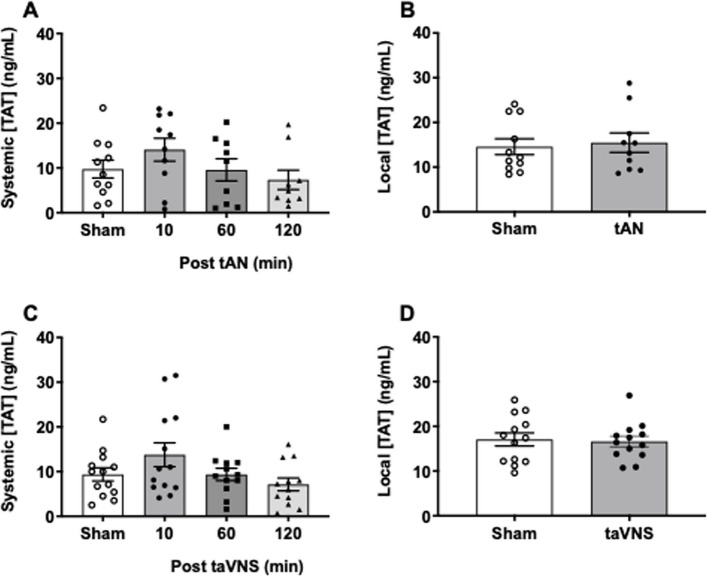
Systemic and local clot production following tAN or taVNS. Circulating blood collected 10 min after sham-stimulation or 10, 60, and 120 min after tAN or taVNS, or local shed blood collected 10 min after sham-stimulation and 10 min after tAN or taVNS, evaluated by thrombin-antithrombin (TAT) complex ELISA. **A**-**B** Systemic and local shed blood TAT concentrations after tAN (*n* = 11). **C**-**D** Systemic and local shed blood TAT concentrations after taVNS (*n* = 13). Error bars represent standard error of the mean. *P* values are compared to post-sham stimulation values. * *p* ≤ 0.05 by repeated measures ANOVA followed by Bonferroni’s multiple comparisons test or by two-tailed paired Student’s t-test

VNS increases local thrombin generation at sites of tissue injury in mice and pigs, whereas systemic thrombin concentrations remain unchanged (Czura et al. 2010; Bravo-Iñiguez et al. 2023). We measured thrombin-antithrombin (TAT) concentrations in circulating blood and locally after finger lancet capillary injury. Compared with sham stimulation, there were no differences in systemic or local TAT concentrations after tAN (Fig. 3A, B). Similarly, there were no differences in systemic or local TAT concentrations after taVNS (Fig. 3C, D). Thus, at these prescribed stimulation settings, neither tAN nor taVNS impacts systemic or local thrombin generation after superficial capillary injury.

### tAN or taVNS facilitates platelet activation

Platelet activation includes secretion of prothrombotic proteins from α and dense granules and conformational change of the primary fibrinogen receptor GPIIb/IIIa (Coller et al. 1983; Harker and Ritchie 1980; Xu et al. 2018). Considering VNS enhances thrombin-mediated platelet α granule secretion in mice, (Bravo-Iñiguez et al. 2023) we first investigated human platelet function with thrombin exposure after tAN or taVNS. Interestingly, compared with sham stimulation, we did not observe any differences in platelet α granule secretion after tAN (Supplemental Fig. 4A) or taVNS (Supplemental Fig. 4B) as quantified by CD62P (P-selectin) expression. For conformational change to the active GPIIb/IIIa receptor, we observed no significant differences in PAC-1 expression after tAN (Supplemental Fig. 4C) or taVNS (Supplemental Fig. 4D). Lastly, we explored platelet dense granule secretion via quantification of CD63. Compared with sham stimulation, we observed significantly lower CD63 expression on platelets one hour after tAN (Supplemental Fig. 4E), but no differences in CD63 expression after taVNS (Supplemental Fig. 4F).

**Fig. 4:**
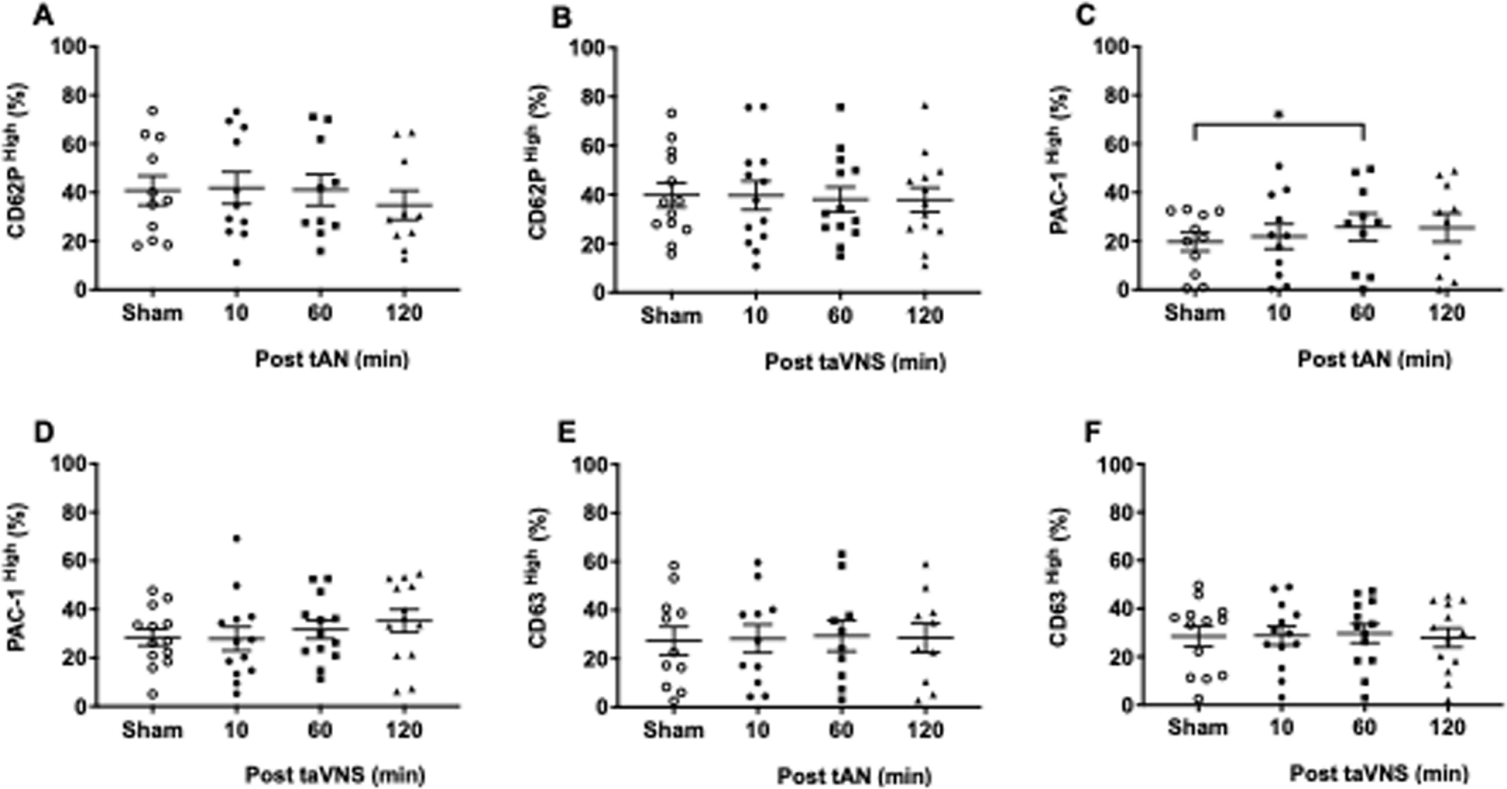
ADP-mediated platelet priming following tAN or taVNS. Peripheral blood platelets collected 10 min after sham-stimulation or 10, 60, and 120 min after tAN or taVNS, were stimulated ex vivo with ADP and evaluated by flow cytometry. **A**-**B** Quantification of surface P-selectin expression (CD62P) from platelet α granule release after tAN (*n* = 11) or taVNS (*n* = 13). **C**-**D** Quantification of active primary fibrinogen receptor GPIIb/IIIa (PAC-1) expression after tAN (*n* = 10) or taVNS (*n* = 13). **E**–**F** Quantification of surface CD63 expression following dense granule release after tAN (*n* = 11) or taVNS (*n* = 13). Error bars represent standard error of the mean. P values are compared to post-sham stimulation values. * *p* ≤ 0.05 by repeated measures ANOVA followed by Bonferroni’s multiple comparisons test

Next, we explored platelet activation with ADP exposure. Compared with sham stimulation, we did not observe any differences in P-selectin expression after tAN (Fig. 4A) or taVNS (Fig. 4B). Compared with sham stimulation, we observed significantly higher active GPIIb/IIIa receptor expression one hour following tAN (sham = 19.7 ± 3.8 vs. tAN = 25.79 ± 5.57; p = 0.035) (Fig. 4C). In contrast, there were no differences in ADP-mediated active GPIIb/IIIa receptor expression at any time-point after taVNS (Fig. 4D). For dense granule secretion, we observed no significant changes in CD63 expression at any time-point following tAN (Fig. 4E) or taVNS (Fig. 4F).

**Fig. 5:**
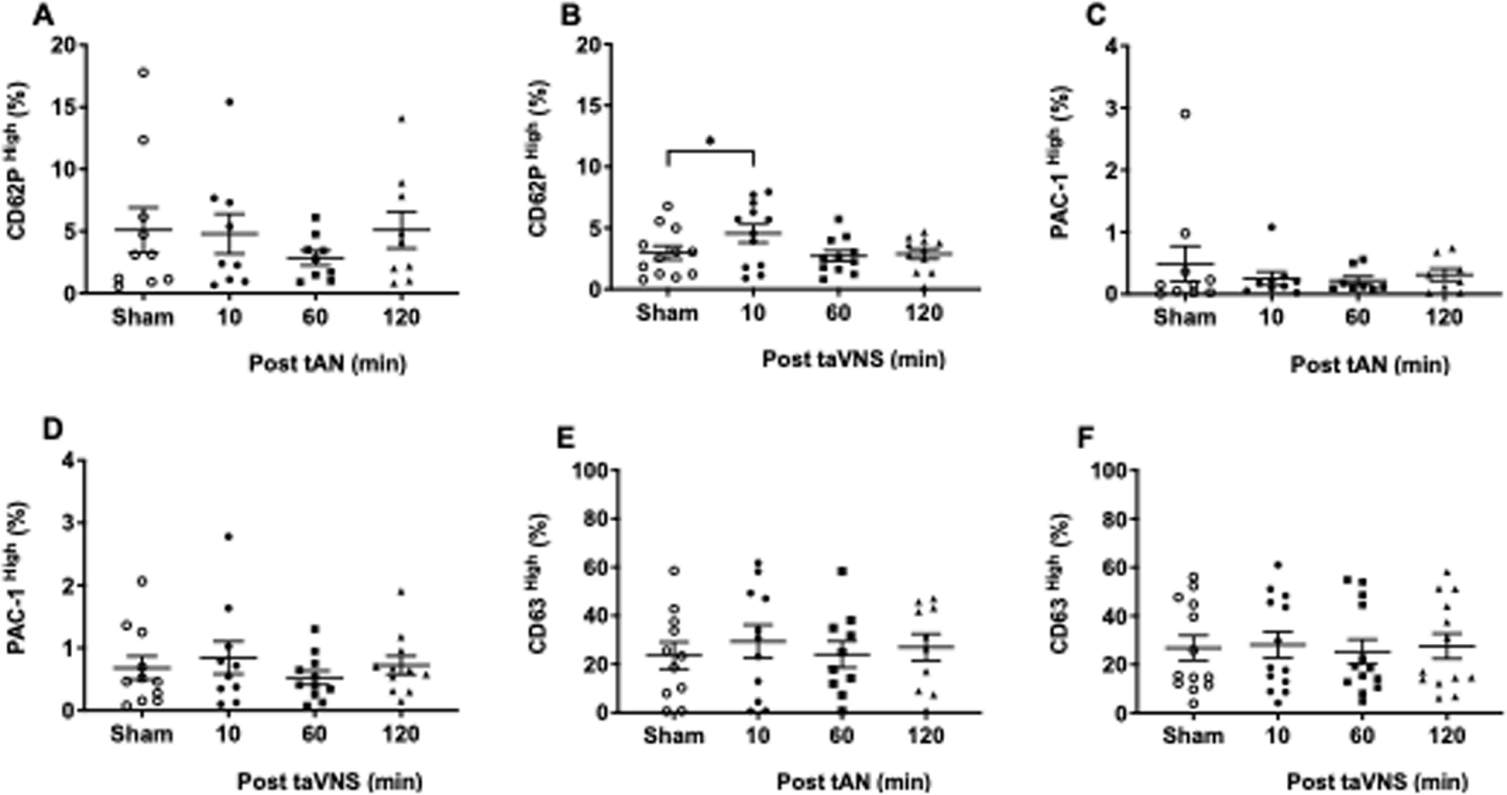
Collagen-mediated platelet priming following tAN or taVNS. Peripheral blood platelets collected 10 min after sham-stimulation or 10, 60, and 120 min after tAN or taVNS, were stimulated ex vivo with collagen and evaluated by flow cytometry. **A**-**B** Quantification of surface P-selectin expression (CD62P) from platelet α granule release after tAN (*n* = 10) or taVNS (*n* = 12). (C-D) Quantification of active primary fibrinogen receptor GPIIb/IIIa (PAC-1) expression after tAN (*n* = 10) or taVNS (*n* = 11). **E**–**F** Quantification of surface CD63 expression following dense granule release after tAN (*n* = 11) or taVNS (*n* = 13). Error bars represent standard error of the mean. *P* values are compared to post-sham stimulation values. * *p* ≤ 0.05 by repeated measures ANOVA followed by Bonferroni’s multiple comparisons test

Lastly, we quantified platelet activation with collagen exposure. Compared with sham stimulation, we observed no changes in P-selectin expression at any time-point after tAN (Fig. 5A), but significantly higher P-selectin expression 10 min after taVNS (sham = 2.97 ± 0.56 vs. taVNS = 4.55 ± 0.75; p = 0.03) (Fig. 5B). For active GPIIb/IIIa receptor expression, we observed no differences at any time-point after tAN (Fig. 5C) or taVNS (Fig. 5D). Finally, compared with sham stimulation, we observed no significant changes in CD63 expression at any time-point following tAN (Fig. 5E) or taVNS (Fig. 5F). Together these findings suggest that tAN primes platelets via enhanced ADP-mediated conformational change to the active GPIIb/IIIa receptor, while taVNS primes platelets via enhanced collagen-mediated α granule secretion.

### taVNS accelerates clot initiation, propagation, and stabilization kinetics

**Fig. 6:**
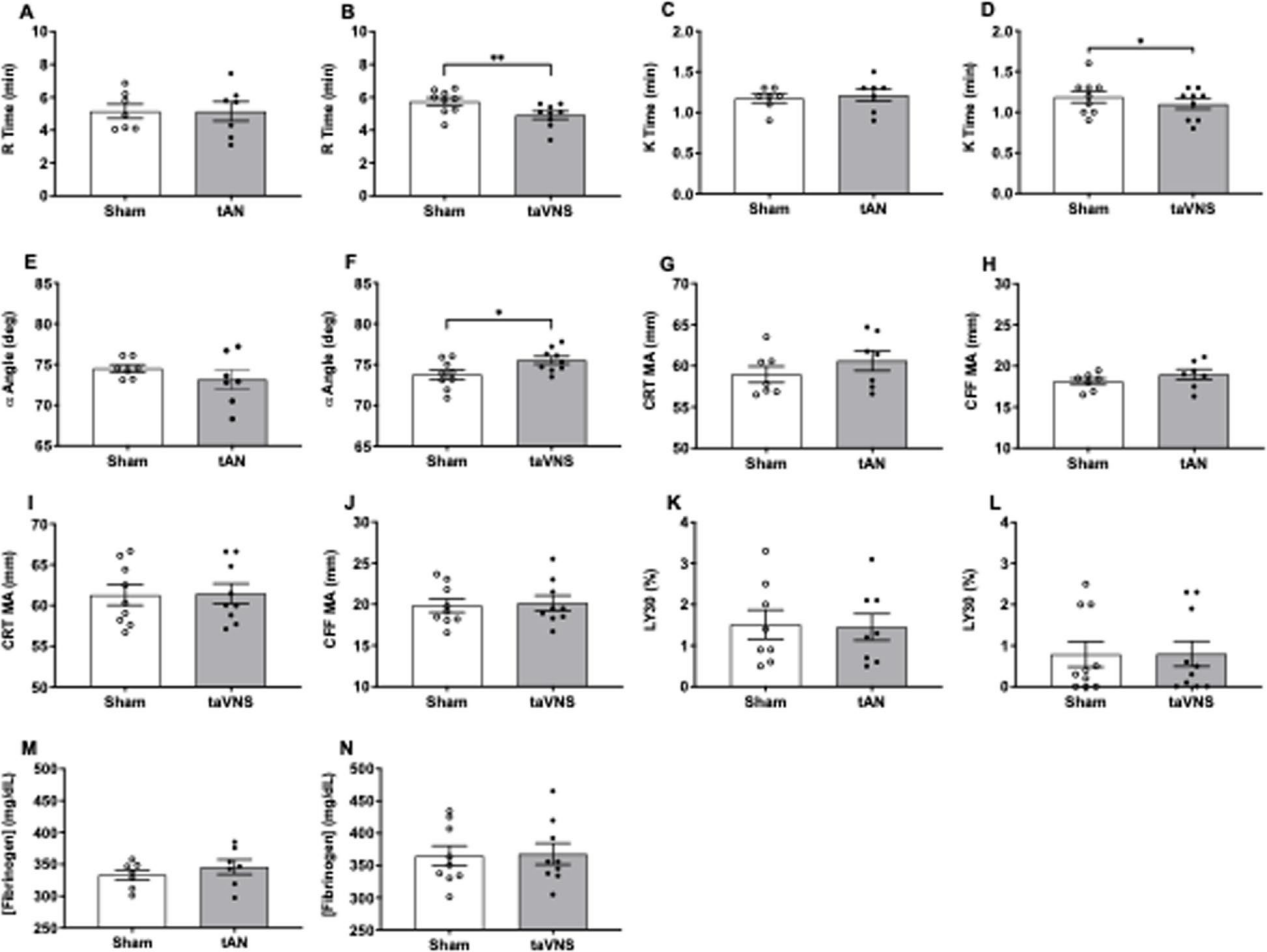
Systemic blood viscoelastic hemostatic profiles following tAN or taVNS. Peripheral circulating blood collected 10 min after sham-stimulation and 10 min after tAN or taVNS, evaluated by thromboelastography (TEG, Haemonetics Inc.). **A**-**B** Time to clot initiation (Reaction, R time) after tAN (*n* = 7) or taVNS (*n* = 9). **C**-**D** Initial speed of clot formation (Kinetical, K time) after tAN (*n* = 7) or taVNS (*n* = 9). **E**–**F** Rate of fibrin cross-linking (α angle) after tAN (*n* = 7) or taVNS (*n* = 9). **G**-**H** Total blood clot strength and firmness secondary to platelets and fibrinogen (Citrated Rapid TEG maximum amplitude) after tAN (*n* = 7) or taVNS (*n* = 9). **I**-**J** Total blood clot strength and firmness secondary to fibrinogen (Citrated Functional Fibrinogen maximum amplitude) after tAN (*n* = 7) or taVNS (*n* = 9). **K**-**L** Percentage of clot fibrinolysis at 30 min (LY30) after tAN (*n* = 8) or taVNS (*n* = 10). **M**–**N** Fibrinogen levels after tAN (*n* = 7) or taVNS (*n* = 9). Error bars represent standard error of the mean. *P* values are compared to post-sham stimulation values. * *p* ≤ 0.05; ** *p* < 0.01 by two-tailed paired Student’s t-test

Thromboelastography (TEG) is a viscoelastic assay that quantifies platelet and coagulation factor contributions to hemostasis (Koh and Hunt 2003). Clinically, TEG evaluates coagulopathic patients at risk for life-threatening hemorrhage, (Matkovic and Lindholm 2022; Perelman et al. 2024) and hypercoagulability associated with systemic thromboembolism (Słomka et al. 2021). We compared TEG readouts from peripheral circulating blood collected 10 min after sham-stimulation versus 10 min after tAN or taVNS. First, we measured R (reaction) time to quantify speed of clot initiation. Compared to sham stimulation, we observed no change in R time following tAN (Fig. 6A), but significantly shorter R time after taVNS (sham = 5.74 ± 0.24 vs. taVNS = 4.91 ± 0.24; p = 0.004) (Fig. 6B). Next, we measured K (kinetical) time to quantify speed of clot propagation. Compared with sham stimulation, we observed no change in K time following tAN (Fig. 6C), but significantly shorter K time following taVNS (sham = 1.189 ± 0.07 vs. taVNS = 1.10 ± 0.06; p = 0.05) (Fig. 6D). Next, we measured α angle, which quantifies rate of clot stabilization. Compared with sham stimulation, there was no change in α angle after tAN (Fig. 6E), but taVNS significantly increased α angle (sham = 73.78 ± 0.57 vs. taVNS = 75.53 ± 0.47; p = 0.02) (Fig. 6F). We then quantified overall clot strength. Citrated rapid teg (CRT) maximum amplitude (MA) quantifies clot strength resulting from platelet and fibrinogen interactions. Citrated functional fibrinogen (CFF) MA incorporates a platelet GPIIb/IIIa receptor inhibitor and represents clot strength resulting only from the contribution of fibrinogen. Compared with sham stimulation, we observed no significant change in CRT MA (Fig. 6G) or CFF MA following tAN (Fig. 6H). Similarly, we did not observe any differences in CRT MA (Fig. 6I) or CFF MA (Fig. 6J) after taVNS. Next, we quantified clot lysis. Compared with sham stimulation, we did not observe any difference in LY30 (% clot lysis at 30 min) after tAN (Fig. 6K) or taVNS (Fig. 6L). Finally, we quantified circulating fibrinogen levels and found no differences after tAN (Fig. 6M) or taVNS (Fig. 6N).

TEG platelet mapping studies elucidate platelet contributions to clot strength via specific intracellular signaling pathways (Chen and Teruya 2009). HKH MA quantifies thrombin-mediated maximal activation of platelets and cleavage of all available fibrinogen (Chen and Teruya 2009). Compared with sham stimulation, we did not observe any differences in HKH MA after tAN (Supplemental Fig. 5A) or taVNS (Supplemental Fig. 6A). ActF MA assesses fibrin’s contribution to clot strength by blocking thrombin (Chen and Teruya 2009). Compared with sham stimulation, we did not observe any differences in ActF MA after tAN (Supplemental Fig. 5B) or taVNS (Supplemental Fig. 6B). ADP MA quantifies how ADP signaling modulates clot strength by blocking thrombin. Compared with sham stimulation, we did not observe any differences in ADP MA after tAN (Supplemental Fig. 5C) or taVNS (Supplemental Fig. 6C). Next, we measured AA MA, which assesses how arachidonic acid (AA) signaling via the thromboxane A2 receptor modulates clot strength by blocking thrombin. Compared with sham stimulation, we did not observe any differences in AA MA after tAN (Supplemental Fig. 5D) or taVNS (Supplemental Fig. 6D). We then determined ADP or AA % aggregation, which calculates MA in response to ADP or AA signaling, and is used clinically to provide insight into the effectiveness of P2Y12 or thromboxane A2 inhibitors, respectively. Compared with sham stimulation, we did not observe any differences in ADP % aggregation after tAN (Supplemental Fig. 5E) or taVNS (Supplemental Fig. 6E). Compared with sham stimulation, we did not observe any differences in AA % aggregation after tAN (Supplemental Fig. 5F) or taVNS (Supplemental Fig. 6F). Lastly, we determined ADP or AA % inhibition, which compares clot strength resulting from ADP or AA pathway activation versus maximal clot strength from all pathways. Compared with sham stimulation, we did not observe any differences in ADP % inhibition after tAN (Supplemental Fig. 5G) or taVNS (Supplemental Fig. 6G). Compared with sham stimulation, we did not observe any differences in AA % inhibition after tAN (Supplemental Fig. 5H) or taVNS (Supplemental Fig. 6H). Together these findings suggest taVNS accelerates clot initiation, speed of clot propagation, and rate of clot stabilization, whereas tAN does not impact kinetics of clot formation, overall strength, or breakdown early after stimulation.

### Safety

The single reported adverse event (AE) occurred during initial blood collection when a participant experienced mild light-headedness that resolved without medical attention. The electrode had been applied to the participant’s ear, but stimulation had not been activated. The AE was deemed unrelated to the intervention.

## Discussion

Invasive VNS primes platelets to accelerate clot formation and improve hemostasis in preclinical hemorrhage models (Czura et al. 2010; Bravo-Iñiguez et al. 2023). Noninvasive tAN reduces menstrual duration and blood loss in women with idiopathic or von Willebrand disease related heavy menstrual bleeding (Czura et al. 2025). Here we showed for the first time that taVNS or tAN primes platelets, the fundamental mammalian cells whose clotting speed and efficiency provide an evolutionary survival advantage (Schmaier et al. 2011). taVNS, but not tAN, also modulated TEG parameters, resulting in accelerated clot initiation, propagation, and stabilization. While these results do not provide direct evidence of improved clinical hemostasis, demonstrating that taVNS and tAN may safely enhance platelet function are important steps to help guide future studies investigating hemorrhage control.

VNS selectively facilitates thrombin-mediated α granule secretion in murine platelets, which correlates with significant reductions in bleeding duration and blood loss after traumatic injury (Bravo-Iñiguez et al. 2023).Interestingly, we found no changes in thrombin-mediated α granule secretion from human platelets. Of note, differential expression or signaling of protease-activated receptors (PAR), which mediate platelet activation via thrombin, has been shown in mice and humans (Renna et al. 2022). Thus, murine and human platelets may respond differently to neural stimulation. ADP enhanced platelet expression of active GPIIb/IIIa receptor after tAN but not taVNS. Collagen enhanced platelet α granule secretion after taVNS but not tAN. Superficial vascular wall injury activates ADP receptor pathways and conformational change of GPIIb/IIIa, as well as platelet glycoprotein VI-collagen interactions, whereas higher-grade wall injury invokes stronger thrombin-dependent platelet responses (Hechler et al. 2010). Thus, at stimulation intensities and durations used here, tAN or taVNS may control less severe bleeding. However, higher intensity and/or longer duration electrical stimulation may activate platelet responses capable of treating more severe injuries. Further studies may elucidate how taVNS and tAN target distinct platelet signaling pathways, and if facilitating collagen- or ADP-mediated platelet activation via taVNS or tAN translates into clinically relevant improvements in hemostasis.

Prolonged clot initiation on TEG suggests quantitative or qualitative coagulation factor deficiency, (Hartmann et al. 2020) whereas shortened R times occur in hypercoagulable states marked by rapid clotting factor activation (Huang et al. 2020; Kataria et al. 2023). In swine hemorrhage, VNS accelerates clot initiation on rotational thromboelastography (RoTEG) by 12% versus sham stimulation, which correlates with a 40% reduction in bleeding duration and nearly 50% reduction in blood loss following injury (Czura et al. 2010). Here we observed that taVNS accelerated TEG clot initiation by 15% versus sham stimulation. In hemophilia A mice, VNS accelerates thrombosis and reduces blood loss by 75% versus sham stimulation (Bravo-Iñiguez et al. 2023). While we did not measure circulating clotting factors here, factor VIII concentrations did not change in hemophilia A mice after VNS (Bravo-Iñiguez et al. 2023). Considering VNS improves swine hemostasis and restores murine hemostasis while bypassing factor VIII deficiency, we reasoned that VNS may facilitate clotting because surface conditions on primed platelets are more favorable to thrombosis. To this end, additional studies should explore whether taVNS can enhance clotting in hemophilia A and other coagulopathies, including vWD or hemophilia B.

In addition to accelerated clot initiation, we observed faster clot propagation and clot stabilization after taVNS. Interestingly, these phenotypic changes were not observed after tAN or VNS in swine (Czura et al. 2010). Clinically, abnormalities of K time or α angle may indicate hypofibrinogenemia. Of note, circulating fibrinogen levels remain unchanged after taVNS. Considering clot formation and strength depend on converting fibrinogen to stable fibrin strands, taVNS may enhance fibrin cross linking via the favorable thrombotic milieu on primed platelets.

Consistent with VNS administration in swine, (Czura et al. 2010) neither tAN nor taVNS regulated clot maximum amplitude. Considering its hemostatic efficacy in women with heavy menstrual bleeding, tAN may modulate bleeding independent of accelerating clotting kinetics. Alternatively, given the shorter tAN duration here, the beneficial effects on TEG may require longer stimulation times. Considering we performed TEG 10 min after tAN, additional time may be necessary to show changes in clotting kinetics. Finally, neither tAN nor taVNS altered clot lysis, suggesting their hemostatic effects hinge on enhanced clot formation. Notwithstanding, combining taVNS or tAN with tranexamic acid, which inhibits clot lysis, might provide a synergistic effect against dysregulated fibrinolysis, particularly during trauma-induced coagulopathy (Moore et al. 2021).

VNS increases thrombin generation at the site of injury in pigs and mice (Czura et al. 2010; Bravo-Iñiguez et al. 2023). Here we found that local thrombin concentrations remain unchanged after tAN or taVNS. This finding may result from dissimilar injury severities. Finger lancets cause dermal capillary injury, whereas preclinical models involved direct arterial or arteriolar disruption. Lastly, neither tAN nor taVNS changed platelet or other blood cell counts. Interestingly, we had similar findings in pigs and mice following VNS (Czura et al. 2010; Bravo-Iñiguez et al. 2023). Considering circulating platelets are stored in the spleen as an exchangeable pool, and VNS targets the spleen to prime platelets, vagal stimulation may optimize qualitative, rather than quantitative, platelet function to enhance hemostasis. However, VNS targets the spleen to regulate leukocyte trafficking to sites of peripheral inflammation, (Huston et al. 2009; Saeed et al. 2005) so it remains plausible that vagal stimulation modulates local platelet numbers at injury sites.

The current study has important limitations. Most study subjects were female, whereas most trauma patients are male. Our sample sizes of 11 and 13 per cohort were relatively small. Our research study coordinator was unblinded to administer the neurostimulation. In many double-blinded trials, research team members with direct subject contact remain blinded. As a small exploratory trial, we did not prespecify primary and secondary outcomes. Because we report multiple outcome measures, there is increased risk of false positives (Type I errors). Therefore, reported p-values should be interpreted with caution. By administering sequential sham and active auricular stimulations, and performing multiple systemic blood draws, all within a short time window, it is plausible that other experimental or physiological factors may have impacted our results. Because we only assessed for adverse events during the short study period, it is not possible to exclude potential late-onset adverse events. There were methodological issues with TEG blood sampling. Considering these occurred randomly and affected treatment groups similarly (36% tAN, 38% taVNS), we excluded them from final analysis, but the missing data may have altered the results. Lastly, because this study involved healthy subjects, and TEG results never deviated from the normal range, it is unclear whether the observed changes in clotting kinetics after taVNS correlate with improved clinical hemostasis. Moreover, there is no standardized methodology to predict changes in clinical hemostasis based on the platelet priming assay. Taken together, additional larger clinical trials are necessary to demonstrate that taVNS or tAN can decrease bleeding in surgery, trauma, post-partum hemorrhage, or bleeding disorders.

## Supplementary Information

Supplemental Figure 1. Platelet gating strategies for flow cytometry. (A) Preliminary FSC/SSC gates for starting platelet cell population from subject whole blood. (B) Platelet cell population identification with anti-CD42b. (C) Platelet gating strategy for anti-CD62P (P-selectin) on X-axis and PAC1 (active GPIIb/IIIa) on Y-axis. (D) Platelet gating strategy for anti-CD63 (phosphatidylserine).

Supplemental Figure 2. Consolidated Standards of Reporting Trials (CONSORT) 2025 Flow Diagram.

Supplemental Figure 3. Circulating blood cell population dynamics following tAN or taVNS. Peripheral circulating blood collected 10 min after sham-stimulation or 10, 60, and 120 min after tAN or taVNS. (A-B) White blood cell counts after tAN (*n *= 11) or taVNS (*n *= 12). (C-D) Hemoglobin levels after tAN (*n *= 11) or taVNS (*n *= 12). (E-F) Hematocrit after tAN (*n *= 11) or taVNS (*n *= 12). (G-H) Platelet counts after tAN (*n *= 10) or taVNS (*n *= 12). Error bars represent standard error of the mean. *P* values are compared to post-sham stimulation values. * *p <* 0.05 by repeated measures ANOVA followed by Bonferroni’s multiple comparisons test.

Supplemental Figure 4. Thrombin-mediated platelet priming following tAN or taVNS. Peripheral blood platelets collected 10 min after sham-stimulation or 10, 60, and 120 min after tAN or taVNS, were stimulated ex vivo with thrombin and evaluated for surface activation marker expression by flow cytometry. (A-B) Quantification of surface P-selectin expression (CD62P) from platelet a granule release after tAN (*n *= 10) or taVNS (*n *= 13). (C-D) Quantification of active primary fibrinogen receptor GPIIb/IIIa (PAC-1) expression after tAN (*n *= 10) or taVNS (*n *= 12). (E-F) Quantification of surface CD63 expression following dense granule release after tAN (*n *= 11) or taVNS (*n *= 13). Error bars represent standard error of the mean. *P* values are compared to post-sham stimulation values. * *p <* 0.05 by repeated measures ANOVA followed by Bonferroni’s multiple comparisons test.

Supplemental Figure 5. TEG platelet mapping following tAN. Peripheral circulating blood collected 10 min after sham-stimulation and 10 min after tAN, evaluated by thromboelastography (TEG, Haemonetics Inc.). (A) Total blood clot strength and firmness secondary to kaolin-induced maximal activation (HKH) after tAN (*n *= 8). (B) Total blood clot strength and firmness secondary to fibrin only with thrombin blockade (ActF) after tAN (*n *= 8). (C) Total blood clot strength and firmness secondary to ADP signaling with thrombin blockade after tAN (n = 8). (D) Total blood clot strength and firmness secondary to arachidonic acid (AA) signaling via thromboxane A2 receptor with thrombin blockade after tAN (*n *= 8). (E) Total % platelet aggregation via ADP signaling after tAN (*n *= 8). (F) Total % platelet aggregation via AA signaling after tAN (*n *= 8). (G) Total % inhibition of platelet aggregation via ADP signaling after tAN (*n *= 8). (H) Total % inhibition of platelet aggregation via AA signaling after tAN (*n *= 8). Error bars represent standard error of the mean. *P* values are compared to post-sham stimulation values. * *p <* 0.05 by two-tailed paired Student’s t-test.

Supplemental Figure 6. TEG platelet mapping following taVNS. Peripheral circulating blood collected 10 min after sham-stimulation and 10 min after taVNS, evaluated by thromboelastography (TEG, Haemonetics Inc.). (A) Total blood clot strength and firmness secondary to kaolin-induced maximal activation (HKH) after taVNS (*n *= 8). (B) Total blood clot strength and firmness secondary to fibrin only with thrombin blockade (ActF) after taVNS (*n *= 8). (C) Total blood clot strength and firmness secondary to ADP signaling with thrombin blockade after taVNS (*n *= 8). (D) Total blood clot strength and firmness secondary to arachidonic acid (AA) signaling via thromboxane A2 receptor with thrombin blockade after taVNS (*n *= 8). (E) Total% platelet aggregation via ADP signaling after taVNS (*n *= 8). (F) Total % platelet aggregation via AA signaling after taVNS (*n *= 8). (G) Total % inhibition of platelet aggregation via ADP signaling after taVNS (*n *= 8). (H) Total % inhibition of platelet aggregation via AA signaling after taVNS (*n *= 8). Error bars represent standard error of the mean. *P* values are compared to post-sham stimulation values. * *p <* 0.05 by two-tailed paired Student’s t-test.

## Data Availability

Deidentified individual participant data that underlie the reported results will be made available 3 months after publication for a period of 5 years after the publication date. Proposals for access should be sent to the corresponding author. The study protocol is included as a data supplement available with the online version of this article.
