# Supplementary figures and images for: Effects of transcutaneous auricular vagus nerve stimulation or combined vagal and trigeminal nerve stimulation on platelet function and laboratory hemostasis parameters in healthy human subjects
Source: Bioelectron Med. 2026 Jun 5;12:14. doi: 10.1186/s42234-026-00208-w (PMC13237925; doi:10.1186/s42234-026-00208-w)

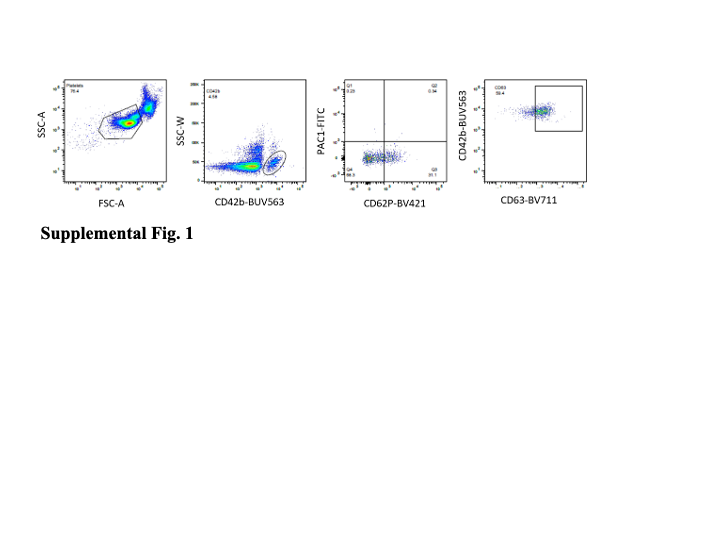

Supplement: Supplementary file 1 — Supplemental Figure 1. Platelet gating strategies for flow cytometry. (A) Preliminary FSC/SSC gates for starting platelet cell population from subject whole blood. (B) Platelet cell population identification with anti-CD42b. (C) Platelet gating strategy for anti-CD62P (P-selectin) on X-axis and PAC1 (active GPIIb/IIIa) on Y-axis. (D) Platelet gating strategy for anti-CD63 (phosphatidylserine). [file 42234_2026_208_MOESM1_ESM.tiff]

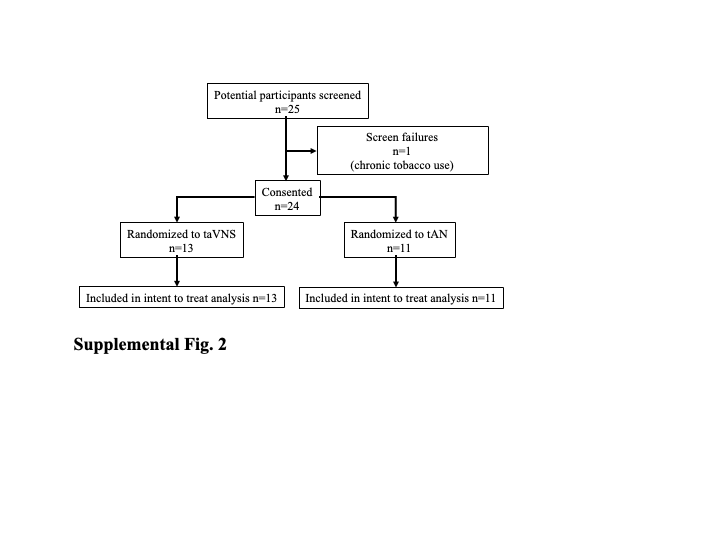

Supplement: Supplementary file 2 — Supplemental Figure 2. Consolidated Standards of Reporting Trials (CONSORT) 2025 Flow Diagram. [file 42234_2026_208_MOESM2_ESM.tiff]

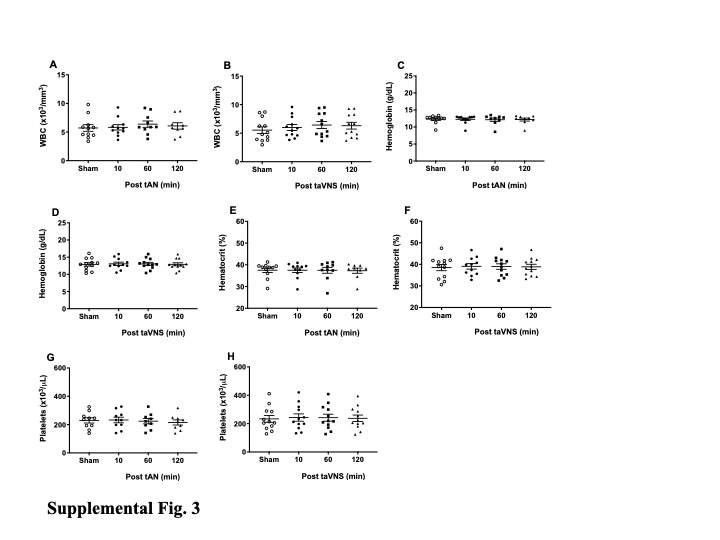

Supplement: Supplementary file 3 — Supplemental Figure 3. Circulating blood cell population dynamics following tAN or taVNS. Peripheral circulating blood collected 10 min after sham-stimulation or 10, 60, and 120 min after tAN or taVNS. (A-B) White blood cell counts after tAN (n = 11) or taVNS (n = 12). (C-D) Hemoglobin levels after tAN (n = 11) or taVNS (n = 12). (E-F) Hematocrit after tAN (n = 11) or taVNS (n = 12). (G-H) Platelet counts after tAN (n = 10) or taVNS (n = 12). Error bars represent standard error of the mean. P values are compared to post-sham stimulation values. * p < 0.05 by repeated measures ANOVA followed by Bonferroni’s multiple comparisons test. [file 42234_2026_208_MOESM3_ESM.tiff]

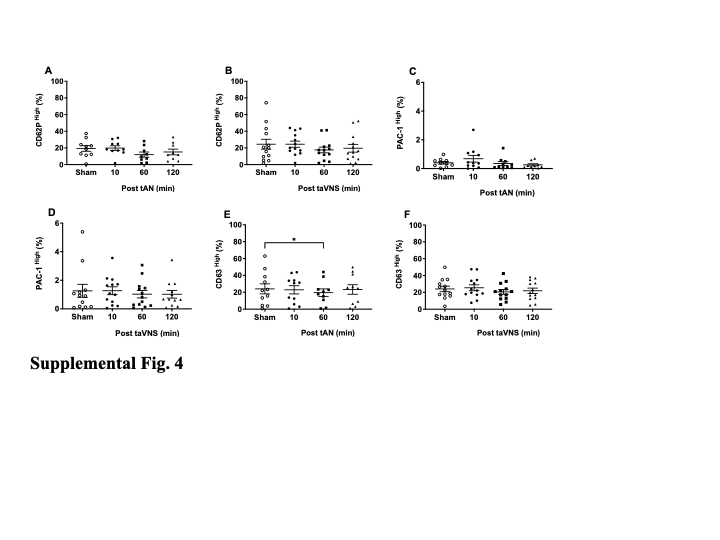

Supplement: Supplementary file 4 — Supplemental Figure 4. Thrombin-mediated platelet priming following tAN or taVNS. Peripheral blood platelets collected 10 min after sham-stimulation or 10, 60, and 120 min after tAN or taVNS, were stimulated ex vivo with thrombin and evaluated for surface activation marker expression by flow cytometry. (A-B) Quantification of surface P-selectin expression (CD62P) from platelet a granule release after tAN (n = 10) or taVNS (n = 13). (C-D) Quantification of active primary fibrinogen receptor GPIIb/IIIa (PAC-1) expression after tAN (n = 10) or taVNS (n = 12). (E-F) Quantification of surface CD63 expression following dense granule release after tAN (n = 11) or taVNS (n = 13). Error bars represent standard error of the mean. P values are compared to post-sham stimulation values. * p < 0.05 by repeated measures ANOVA followed by Bonferroni’s multiple comparisons test. [file 42234_2026_208_MOESM4_ESM.tiff]

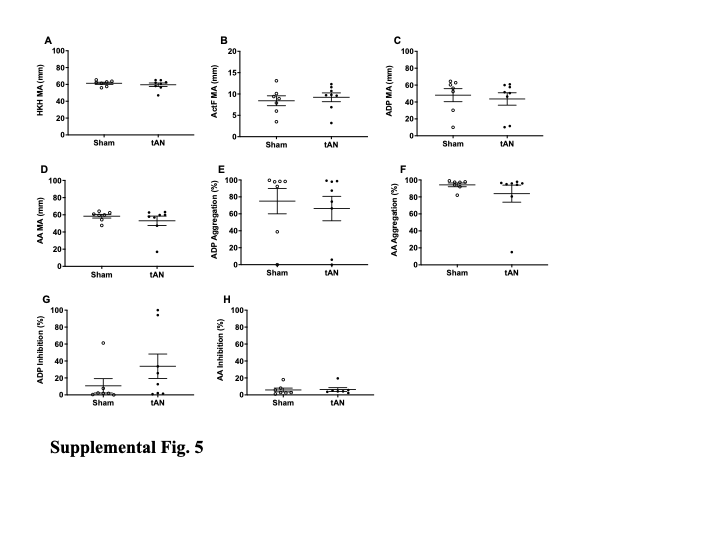

Supplement: Supplementary file 5 — Supplemental Figure 5. TEG platelet mapping following tAN. Peripheral circulating blood collected 10 min after sham-stimulation and 10 min after tAN, evaluated by thromboelastography (TEG, Haemonetics Inc.). (A) Total blood clot strength and firmness secondary to kaolin-induced maximal activation (HKH) after tAN (n = 8). (B) Total blood clot strength and firmness secondary to fibrin only with thrombin blockade (ActF) after tAN (n = 8). (C) Total blood clot strength and firmness secondary to ADP signaling with thrombin blockade after tAN (n = 8). (D) Total blood clot strength and firmness secondary to arachidonic acid (AA) signaling via thromboxane A2 receptor with thrombin blockade after tAN (n = 8). (E) Total % platelet aggregation via ADP signaling after tAN (n = 8). (F) Total % platelet aggregation via AA signaling after tAN (n = 8). (G) Total % inhibition of platelet aggregation via ADP signaling after tAN (n = 8). (H) Total % inhibition of platelet aggregation via AA signaling after tAN (n = 8). Error bars represent standard error of the mean. P values are compared to post-sham stimulation values. * p < 0.05 by two-tailed paired Student’s t-test. [file 42234_2026_208_MOESM5_ESM.tiff]

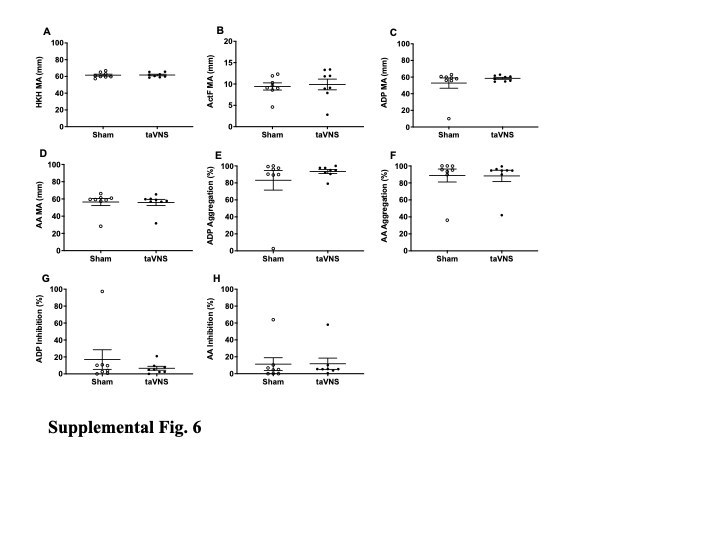

Supplement: Supplementary file 6 — Supplemental Figure 6. TEG platelet mapping following taVNS. Peripheral circulating blood collected 10 min after sham-stimulation and 10 min after taVNS, evaluated by thromboelastography (TEG, Haemonetics Inc.). (A) Total blood clot strength and firmness secondary to kaolin-induced maximal activation (HKH) after taVNS (n = 8). (B) Total blood clot strength and firmness secondary to fibrin only with thrombin blockade (ActF) after taVNS (n = 8). (C) Total blood clot strength and firmness secondary to ADP signaling with thrombin blockade after taVNS (n = 8). (D) Total blood clot strength and firmness secondary to arachidonic acid (AA) signaling via thromboxane A2 receptor with thrombin blockade after taVNS (n = 8). (E) Total% platelet aggregation via ADP signaling after taVNS (n = 8). (F) Total % platelet aggregation via AA signaling after taVNS (n = 8). (G) Total % inhibition of platelet aggregation via ADP signaling after taVNS (n = 8). (H) Total % inhibition of platelet aggregation via AA signaling after taVNS (n = 8). Error bars represent standard error of the mean. P values are compared to post-sham stimulation values. * p < 0.05 by two-tailed paired Student’s t-test. [file 42234_2026_208_MOESM6_ESM.tiff]
